# Early-TIPS Versus Current Standard Therapy for Acute Variceal Bleeding in Cirrhosis Patients: A Systemic Review With Meta-analysis

**DOI:** 10.3389/fphar.2020.00603

**Published:** 2020-05-20

**Authors:** Shuang Li, Chao Zhang, Lu-Lu Lin, Qi Wang, Hong-Xia Zuo, Ai-Ling Zhan, Jie Luo, Yu-Ming Niu, Guo-Qing Zhong

**Affiliations:** ^1^Center for Evidence-Based Medicine and Clinical Research, Taihe Hospital, Hubei University of Medicine, Shiyan, China; ^2^Department of Anesthesiology, Central Hospital of Shanghai Songjiang District, Shanghai, China; ^3^Department of Obstetrical, Jining No.1 People's Hospital, Jining, China

**Keywords:** acute variceal bleeding, cirrhosis, transjugular intrahepatic portosystemic shunt, endoscopic variceal ligation, meta-analysis

## Abstract

**Background:**

The survival of early placement (within 72h after admission) of transjugular intrahepatic portosystemic shunts (early-TIPS) in patients with cirrhosis and acute variceal bleeding (AVB) is controversial.

**Objectives:**

We performed a systemic review and meta-analysis to assess whether early-TIPS could improve survival in patients with cirrhosis and acute variceal bleeding.

**Methods:**

A systematic search of the literature was conducted in PubMed, EMBASE, and Cochrane Library published before 25 June 2019 for eligible studies that compared early-TIPS with a combination of endoscopic variceal ligation (EVL) and pharmacotherapy in the therapeutic effect in AVB patients.

**Results:**

A total of five studies with 1,754 participants were enrolled. The early-TIPS demonstrated a significant improvement in prevention of treatment failure (OR=0.11,95%CI=0.05-0.23), 6-weeks mortality (OR=0.24,95%CI=0.13-0.46), rebleeding within 6 weeks (OR=0.21,95%CI=0.12-0.36), rebleeding within 1 year (OR=0.16,95%CI=0.07-0.36), new or worsening ascites (OR=0.33,95%CI=0.21-0.53), except in encephalopathy (OR=1.29,95%CI=0.996-1.67). For 1-year mortality, a significant prior effect was also observed in early-TIPS (OR=0.64,95%CI=0.46-0.90), and the beneficial effect in Child-Pugh C patients (OR=0.35,95%CI=0.18-0.68) was equal to Child-Pugh B patients (OR=0.34,95%CI=0.25-0.58). No difference in liver transplantation and mortality caused by liver failure was observed.

**Conclusions:**

Early covered-TIPS could be recommended for the management of AVB patients in cirrhosis demonstrating a significant improvement in treatment failure, both short- and long-term mortality, rebleeding risk, and new or worsening ascites compared to standard therapy, especially for high-risk AVB patients. It will also apply to patients with Child-Pugh A until solutions to prevent hepatic encephalopathy in future research are found.

## Introduction

Acute variceal bleeding (de Franchis, 2015) (AVB) is a severe and emergency complication associated with a 20% mortality at 6 weeks in patients with advanced cirrhosis. As the most life-threatening complication in cirrhosis patients, this medical field has received significant attention. Over the past few decades, the recommended standard treatment for patients with AVB involves a combination of endoscopic therapy, vasoactive drugs, and antibiotic therapy (Garcia-Tsao and Bosch, 2010; Tripathi et al., 2015; Garcia-Tsao et al., 2017). Nevertheless, the curative effects are not as good as we expected, including treatment failure occurring in 10-20% of patients, an inevitable risk of rebleeding within the first 48–72 hours, and over 50% of rebleeding episodes occurring within the first 10 days required further rescue therapy—especially in patients with high risk of treatment failure or rebleeding (patients with Child-Pugh C or Child-Pugh B with acute bleeding on endoscopy) (de Franchis, 2015; Njei et al., 2017).

A transjugular intrahepatic portosystemic shunt (TIPS) procedure is a minimally invasive, image-guided intervention used for the prevention of rebleeding and as salvage therapy in patients with refractory variceal hemorrhage, instead of first-line therapy (LaBerge et al., 1993; Rossle et al., 1994). However, studies show that worsening liver function occurred in those patients, which remains high (27%-55%) and also plays a key role as a predictive factor in poor prognosis such as sepsis, hepatic encephalopathy, shock, and death (Chau et al., 1998; Azoulay et al., 2001; Bosch, 2001; Trebicka, 2018). In recent years, research (Monescillo et al., 2004; Garcia-Pagan et al., 2010; Garcia-Pagan et al., 2013; Rudler et al., 2014; Bucsics et al., 2017; Lv et al., 2019a; Lv et al., 2019b) aimed at exploring whether early-TIPS (placed within 72 hours after esophagogastroduodenoscopy (EGD) or endoscopic variceal ligation (EVL)) could replace standard therapy (EVL *plus* NSBB *plus* ANTIBIOTICS) as first-line therapy in AVB is increased. Several RCTs (Moher et al., 2009; Garcia-Pagan et al., 2010) demonstrated a significantly lower rebleeding rate, with no change in mortality, and a higher incidence of hepatic encephalopathy in patients who received TIPS. The 2017 Practice Guidance (Garcia-Tsao et al., 2017) by the American Association for the Study of Liver Diseases (AASLD) also recommend that the early-TIPS procedure should be placed in patients with a high risk of treatment failure or rebleeding. Nevertheless, whether early-TIPS could reduce the mortality and risk of hepatic encephalopathy remains controversial. There has only been one meta-analysis (Deltenre et al., 2015) in 2015 which did not report on the short-term (6 weeks) mortality and rebleeding risk. Therefore, new research revealing early-TIPS, with its current standard therapy, is necessary and beneficial for clinical practice.

In this study, we performed a systemic review with a meta-analysis evaluating whether early-TIPS should be the first-line therapy for current standard care of AVB in cirrhosis patients.

## Methods

### Data Sources and Literature Searches

This systemic review and meta-analysis was based on Preferred Reporting Items for Systematic Reviews and Meta-Analysis guidelines (PRISMA) (Moher et al., 2009) and conducted using the Cochrane Collaboration's systematic review framework (Higgins and G, 2011). PubMed, EMBASE, and the Cochrane Library were searched up until 25 June 2019 for eligible studies investigating early-TIPS versus pharmacotherapy, EVL, and combination therapy in patients with AVB, using the following MeSH words and key terms: “esophageal varices”, “variceal rebleeding”, “variceal hemorrhage”, “portal hypertension”, “liver cirrhosis”, “pharmacotherapy”, “endoscopic variceal ligation”, “TIPS”, “early-TIPS”, and “transjugular intrahepatic portosystemic shunt”.

### Literature Selection and Exclusion

Studies were included in this systemic review and meta-analysis if they met the following criteria: (1) Patients over 16 years old with acute esophageal varices in cirrhosis (or combined gastric varices, but not gastric varices only); (2) PTFE-covered TIPS was placed within 72h after the index bleeding or esophagogastroduodenoscopy or endoscopic therapy; (3) RCTs and non-RCTs compared early-TIPS with standard therapy (defined as a combination of pharmacotherapy *plus* endoscopic therapy). The primary outcomes were defined as the treatment failure (defined as refractory bleeding or rebleeding within 5 days), short-term (during 6-weeks follow up) all-cause mortality and rebleeding risk, and risk of hepatic encephalopathy. Secondary outcomes were defined as the long-term (during 1-year follow up) all-cause mortality and rebleeding risk, risk of new or worsening ascites, rate of liver transplantation, and risk of mortality caused by rebleeding.

Exclusion criteria were the following: (1) TIPS was placed as a rescue therapy; (2) patients with hepatocellular carcinoma (HCC) that did not meet the Milano criteria (Mazzaferro et al., 1996) for transplantation; (3) a history of previous use of a portosystemic shunt or TIPS; (4) bleeding from isolated gastric or ectopic varices; (5) patients with total portal-vein thrombosis; (6) patients > 75 years; (7) patients with severe systemic disease (renal failure, heart failure, etc.).

### Data Extraction

Two independent reviewers extracted relevant data from the eligible studies. When it came to disagreements, consultation was carried out with a third reviewer. The relevant data consists of study design, patient characteristics, interventions, controls, and outcomes.

### Quality Assessment of Included Studies

The Newcastle-Ottawa Scale (NOS) was used to evaluate the methodological quality of non-RCTs studies by two independent commentators. Studies that achieved six or more stars on the modified NOS were considered high quality (Stang, 2010). To evaluate risk of bias for RCTs by two independent commentators, the Cochrane Collaboration tool (Higgins et al., 2011) for assessment of bias was performed, which considers seven domains including adequacy of sequence generation, allocation concealment, blinding of participants, blinding of outcome assessment, incomplete outcome data, selective outcome reporting, and other potential sources of bias, and each item was graded as “high risk”, “low risk”, or “unclear”.

### Statistical Analysis

The dichotomous outcomes (Higgins and G, 2011) were expressed as the odds ratio (OR) with a 95% confidence interval (CI). Heterogeneity (Higgins and Thompson, 2002) between studies was assessed by the I^2^ statistic, in which the significance level was set to P <0.1. To qualify the inconsistency between studies, when I^2^ <40%, we considered that the heterogeneity was mild, then a fixed effect model was performed. However, considering the possibility of heterogeneity between studies, when I^2^ >40%, the heterogeneity was considered non-negligible, therefore, the random effect model was performed instead of the fixed effect model.

Based on the diversity of studies, it is unavoidable to observe the heterogeneity in outcomes. Therefore, a subgroup analysis was conducted by variate of Child-Pugh class (Child-Pugh B and Child-Pugh C) and study design (RCTs and non-RCTs) to explore the source of heterogeneity. For studies of population enrolled patients with Child-Pugh A, which are not considered suitable participants in the AASLD guideline (Garcia-Tsao et al., 2017), we excluded these studies in primary outcomes for sensitivity analysis to detect the stability of the results. In addition, the Egger's test (Egger et al., 1997) was conducted to detect potential publication bias, and all the statistical analyses were performed by Stata 12.0.

## Results

### Characteristics of Eligible Studies

**Figure 1:**
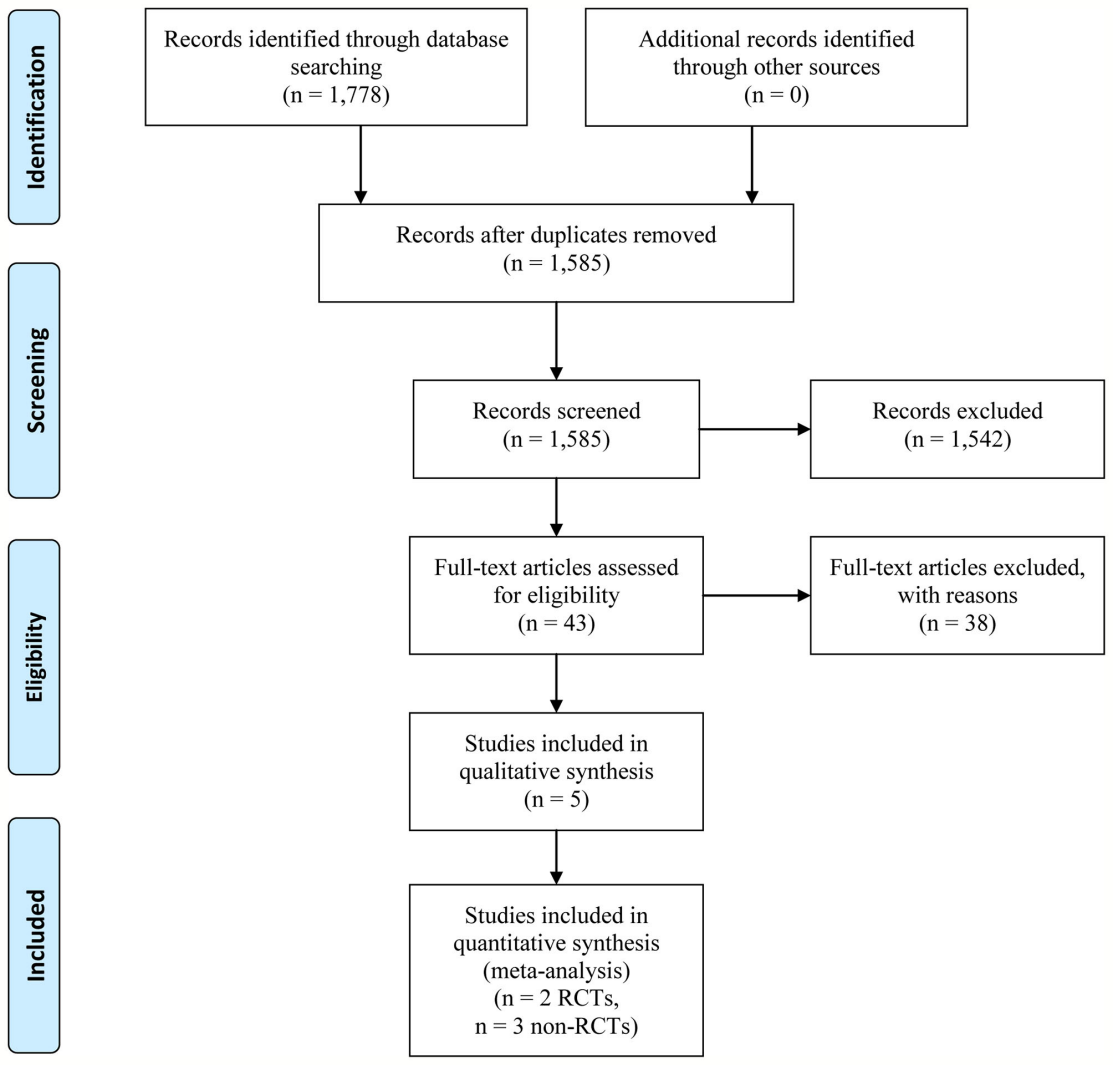
Selection process of included studies.

Our systematic literature search identified 1,778 potential publications. Based on the selection criteria, independent reviewers obtained quantitative data for our meta-analysis by reading all titles, abstracts, and full text evaluations. Eventually, five studies (Garcia-Pagan et al., 2010; Garcia-Pagan et al., 2013; Rudler et al., 2014; Lv et al., 2019a; Lv et al., 2019b) were included with 1,754 (398 of early-TIPS and 1,356 of standard therapy) enrolled participants, two of them were RCTs (Garcia-Pagan et al., 2010; Lv et al., 2019a), and the other three were non-RCT (Garcia-Pagan et al., 2013; Rudler et al., 2014; Lv et al., 2019b) (**Figure 1**). The characteristics of each individual study are presented in **Table 1**. The reasons for exclusion of literature are presented in **Supplementary Table 1**.

**Table 1:** Characteristics of included studies.

References	Study design	Mean age (mean)*	Gender	Etiology of cirrhosis^*^			Child-Pugh class^*^			Standard/Early-TIPS	Type of TIPS	Follow-up (month)*
			Male/Female	Alcohol	Viral	Total	A	B	C			
García -Pagán, 2010	RCT	49/52	44/19	20/22	5/4	31/32	0/0	16/16	15/16	Pharmacotherapy *plus* EVL; TIPS was performed within 72 hours	Covered	10.6/14.6
García -Pagán, 2013	Non-RCT	55/56	52/21	18/25	4/5	30/45	0/0	10/18	20/27	Pharmacotherapy *plus* EVL; TIPS was performed within 72 hours	Covered	14.6/13.1
Rudler et al., 2014	Non-RCT	52.4/53.2	49/13	77/77	3/7	31/31	NA	NA	NA	Pharmacotherapy *plus* EVL; TIPS were performed within 72 hours	Covered	7.8
Lv et al., 2019a	Non-RCT	52/54	984/441	123/14	743/ 133	1219/ 206	455/ 40	654/ 131	88/33	Pharmacotherapy *plus* EVL; TIPS was performed within 72 hours	Covered	23.4/22.9
Lv et al., 2019b	RCT	50.9/50.7	87/42	4/2	38/65	45/84	0/0	35/65	10/19	Pharmacotherapy *plus* EVL; TIPS was placed within 72 hours	Covered	24/24

*Data was expressed as standard therapy versus early-TIPS. TIPS, transjugular intrahepatic portosystemic shunt; NA, not available; EVL, endoscopic variceal ligation; RCT, randomized controlled trail.

### Quality of Included Studies

Three cohort studies (Garcia-Pagan et al., 2013; Rudler et al., 2014; Bucsics et al., 2017) were assessed by NOS (Stang, 2010), the methods for determining exposure factors were reasonable, however, there were some patients in the studies who suffered hepatic encephalopathy at the start, which was considered a primary outcome in our studies. In addition, the other item did not miss the score, therefore, the scores of all studies were more than six as shown in **Supplementary Table 2**, available online.

The Cochrane risk of bias tool (Higgins et al., 2011) demonstrated no high bias in two included RCTs (Garcia-Pagan et al., 2010; Lv et al., 2019a), and the outcomes are shown in **Supplementary Table 3**, available online.

### Primary Outcomes

#### Treatment Failure

**Figure 2:**
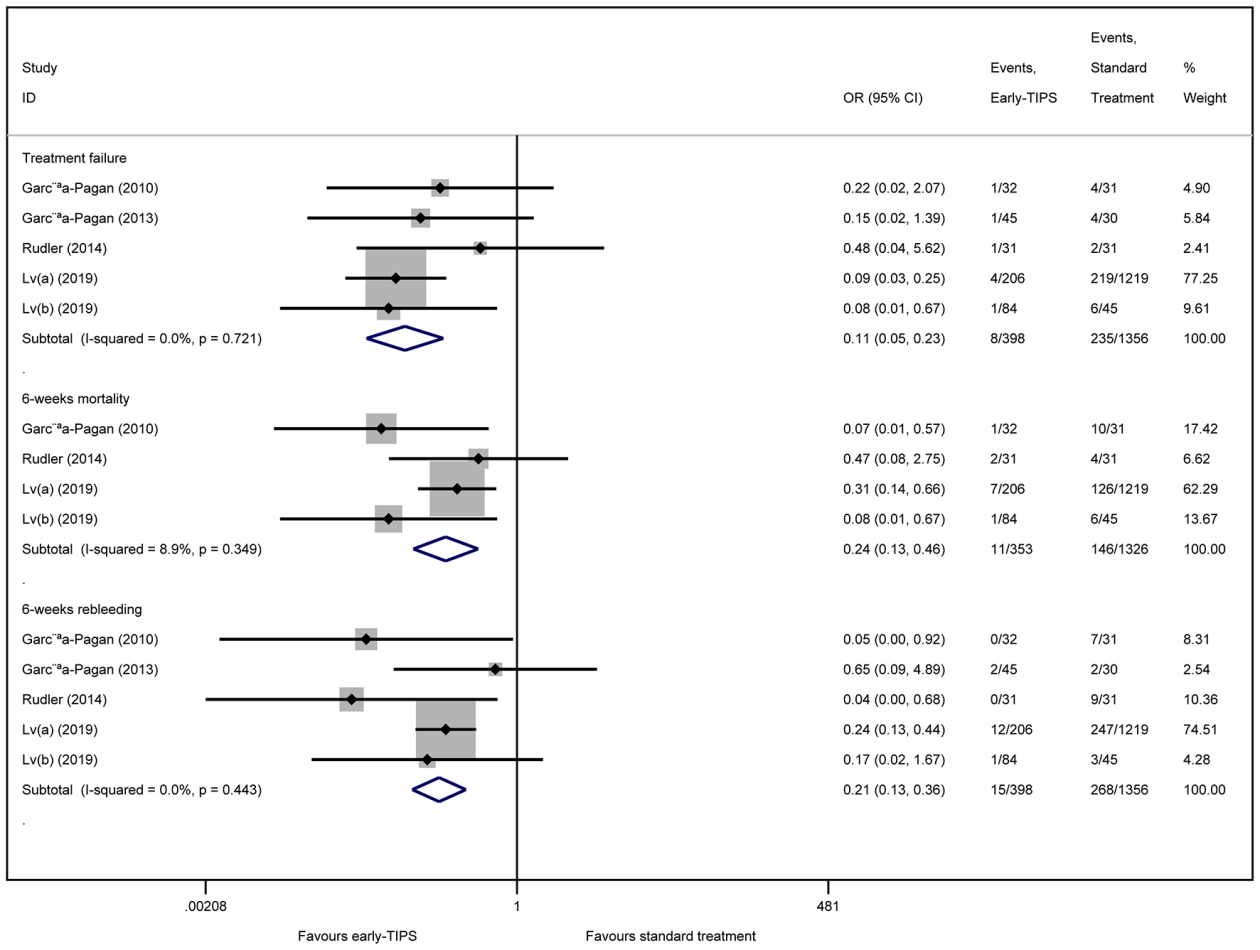
Forest plot demonstrating treatment failure, short-term mortality (6-weeks) and short-term rebleeding risk (6-weeks).

Five studies (Garcia-Pagan et al., 2010; Garcia-Pagan et al., 2013; Rudler et al., 2014; Lv et al., 2019a; Lv et al., 2019b) reported this outcome with eight (2.0%) in the early-TIPS group and 235 (17.3%) in the standard treatment group. As expected, early-TIPS showed a statistical significance to prevent treatment failure in patients with a low statistic difference (OR=0.11, 95%CI= 0.05-0.23, P<0.001; I^2^ = 0%, P=0.721) shown in **Figure 2**.

Subgroup analysis shown in **Table 2** demonstrated a significant reduction in RCTs (OR=0.13, 95%CI=0.03-0.58, P=0.008; I^2^ = 0%, P=0.520), and non-RCTs (OR=0.11, 95%CI=0.04-0.25, P<0.001; I^2^ = 0%, P=0.437).

**Table 2:** The results of subgroup analysis in all outcomes.

Outcomes	Studies (n)	Early-TIPS/ Standard	Percentage (%)	OR	95%CI	P for OR	I^2^ (%)	P for I^2^
**Treatment failure**	5	8/235	2.0/17.3	0.11	0.05-0.23	<0.001	0	0.721
RCTs	2	2/10	17.2/13.2	0.13	0.03-0.58	0.008	0	0.520
Non-RCTs	3	6/225	2.1/16.6	0.11	0.04-0.25	<0.001	0	0.437
**6-weeks mortality**	4	11/146	3.1/11.0	0.24	0.13-0.46	<0.001	8.9	0.349
RCTs	2	2/16	1.7/21.1	0.07	0.02-0.33	0.001	0	0.925
Non-RCTs	2	9/130	3.8/10.4	0.32	0.16-0.65	0.002	0	0.667
**6-weeks rebleeding**	5	15/268	3.8/19.8	0.21	0.12-0.36	<0.001	0	0.443
RCTs	2	1/10	0.9/13.2	0.09	0.02-0.53	<0.001	0	0.507
Non-RCTs	3	14/258	5.0/20.2	0.23	0.13-0.40	<0.001	21.7	0.279
**Hepatic encephalopathy**	5	151/392	15.1/18.6	1.29	0.996-1.67	0.054	35.4	0.185
RCTs	3	37/28	31.9/36.7	0.78	0.42-1.45	0.438	0	0.377
Non-RCTs	3	114/364	40.4/28.4	1.16	1.08-1.90	0.012	14.7	0.309
**1-year mortality**	5	60/253	15.1/18.6	0.64	0.46-0.90	0.010	38.4	0.165
Child-Pugh B	4	33/297	20/43.7	0.34	0.25-0.58	<0.001	0	0.470
Child-Pugh C	4	17/56	22.4/44.5	0.35	0.18-0.68	0.002	10.1	0.329
RCTs	2	16/24	13.8/40.2	0.36	0.17-0.73	0.005	0	0.374
Non-RCTs	3	44/229	15.6/17.9	0.75	0.52-1.08	0.122	27.2	0.253
**1-year rebleeding**	5	34/442	8.5/32.6	0.16	0.07-0.36	<0.001	52.7%	0.076
RCTs	2	9/22	7.8/28.9	0.17	0.02-1.56	0.116	73.3	0.053
Non-RCTs	3	25/420	8.9/31.7	0.12	0.39-0.40	<0.001	57.1	0.097
**Bleeding-related mortality**	4	1/95	0.3/7.2	0.07	0.02-0.29	<0.001	0	0.989
RCTs	1	0/5	0/16.1	0.07	0.004-1.40	0.083	NA	NA
Non-RCTs	3	1/90	0.4/7.0	0.07	0.02-0.33	0.001	0	0.940
**Mortality by liver failure**	5	33/82	8.3/6.0	1.33	0.84-2.12	0.222	1	0.400
RCTs	2	6/5	5.2/6.6	0.71	0.21-2.38	0.580	7.2	0.299
Non-RCTs	3	27/77	9.6/6.0	1.50	0.91-2.46	0.111	13.6	0.314
**Liver transplantation**	5	36/88	9.0/6.5	1.36	0.86-2.15	0.183	0	0.891
RCTs	2	6/3	5.2/3.9	1.66	0.39-6.94	0.491	0	0.668
Non-RCTs	3	30/85	10.6/6.6	1.33	0.82-2.16	0.247	0	0.652
**Ascites (new/worsening)**	4	33/158	9.0/11.9	0.33	0.21-0.53	<0.001	0	0.445
RCTs	2	19/29	16.4/38.2	0.30	0.15-0.60	0.001	0	0.431
Non-RCTs	2	14/129	5.6/10.3	0.30	0.12-0.76	0.001	49.6%	0.159

TIPS, transjugular intrahepatic portosystemic shunt; RCT, randomized controlled trail; NA, not available; RR, relative risk; CI, confidence interval.

### Short-Term Mortality (6 Weeks)

Four studies (Garcia-Pagan et al., 2010; Rudler et al., 2014; Lv et al., 2019a; Lv et al., 2019b) reported this outcome with a total of 11 (3.1%) patients receiving early-TIPS versus 146 (11.0%) receiving standard therapy and who died at 6 weeks (OR=0.24, 95%CI= 0.13-0.46, P<0.001; I^2^ = 8.9%, P=0.349) in **Figure 2**, which demonstrated a significant improvement on 6 weeks survival in early-TIPS.

A subgroup analysis in **Table 2** by study type demonstrated a significant decrease in non-RCTs (OR=0.32, 95%CI=0.16-0.65, P=0.002; I^2^ = 0%, P=0.667), and RCTs (OR=0.07, 95%CI=0.02-0.33, P=0.001; I^2^ = 0%, P=0.925).

#### Short-Term Recurrent Bleeding (6 Weeks)

Five studies (Garcia-Pagan et al., 2010; Garcia-Pagan et al., 2013; Rudler et al., 2014; Lv et al., 2019a; Lv et al., 2019b) reported this outcome. A total of 15 (3.8%; in early-TIPS) and 268 (19.8%; in standard treatment) patients had recurrent bleeding between 5 days and 6 weeks. A significant greater effect on preventing rebleeding at 6 weeks was obtained in early-TIPS (OR=0.21, 95%CI= 0.12-0.36, P<0.001; I^2^ = 0%, P=0.443) in **Figure 2**.

A subgroup analysis in **Table 2** demonstrated a significant reduction in RCTs (OR= 0.09, 95%CI=0.02-0.53, P<0.001; I^2^ = 0%, P=0.507) and non-RCTs (RR=0.23, 95%CI=0.13-0.40, P<0.001, I^2^ = 21.7%; P=0.279).

#### Hepatic Encephalopathy

Five studies (Garcia-Pagan et al., 2010; Garcia-Pagan et al., 2013; Rudler et al., 2014; Lv et al., 2019a; Lv et al., 2019b) reported this outcome with an overall 151 (15.1%) and 392 (18.6%) patients suffering hepatic encephalopathy in early-TIPS and standard therapy, respectively. However, there was no connection observed for increasing risk of hepatic encephalopathy for the early-TIPS group, compared to standard therapy (OR=1.29, 95%CI= 0.996-1.67, P=0.054; I^2^ = 35.4%, P=0.185) in **Figure 4**.

A subgroup analysis in **Figure 2** demonstrated no statistically significant difference in RCTs (OR=0.78, 95%CI=0.42-1.45, P=0.438; I^2^ = 0%, P=0.377). Nevertheless, an increasing risk was observed in non-RCTs (OR=1.16, 95%CI=1.08-1.90, P=0.012; I^2^ = 14.7%, P=0.309).

### Secondary Outcomes

#### Long-Term Mortality (1 Year)

**Figure 3:**
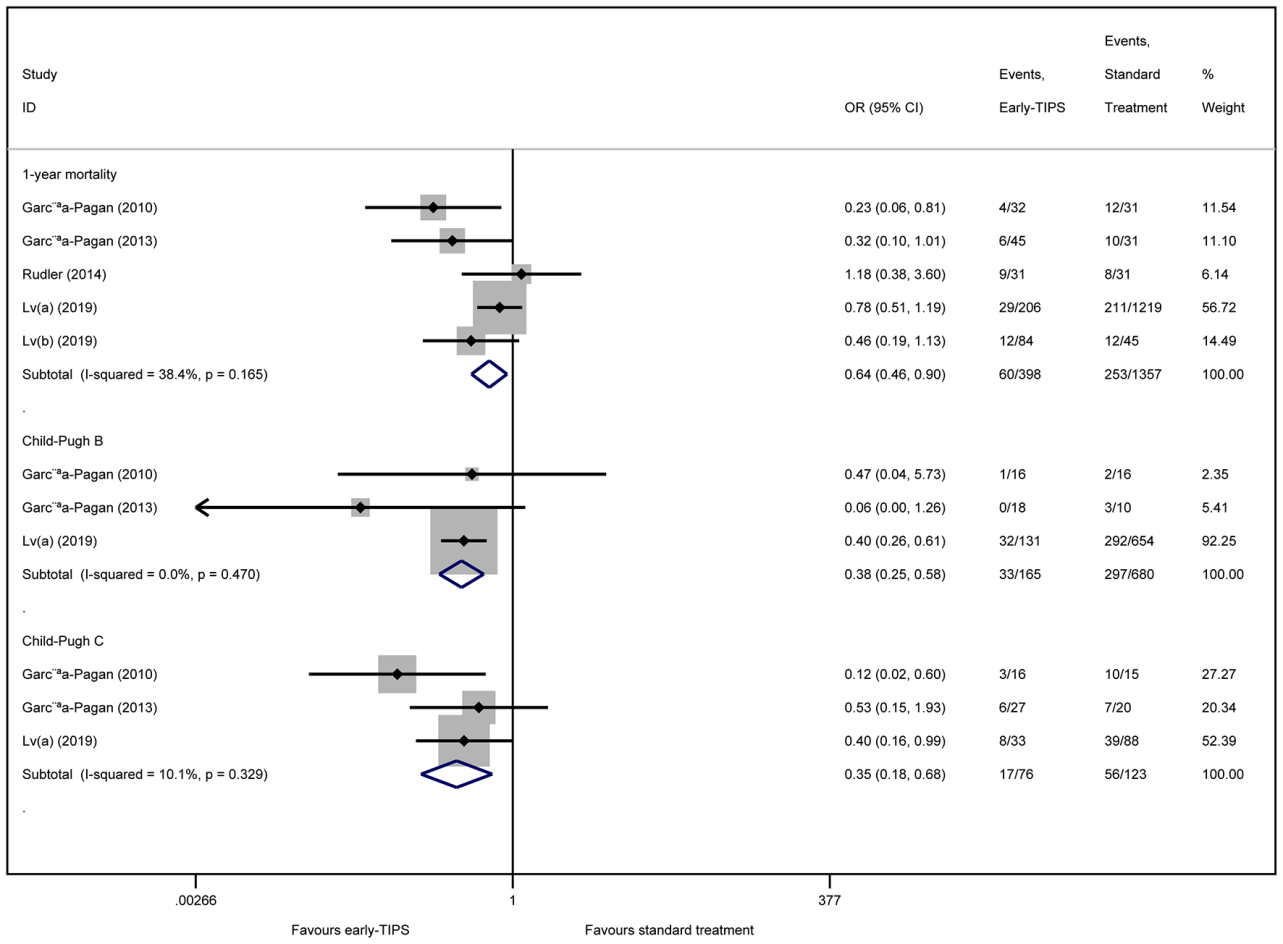
Forest plot demonstrating long-term mortality (1-year) and subgroup analysis according to Child-Pugh class.

Five studies (Garcia-Pagan et al., 2010; Garcia-Pagan et al., 2013; Rudler et al., 2014; Lv et al., 2019a; Lv et al., 2019b) reported this outcome with an overall 60 (15.1%) and 253 (18.6%) patients who died during 1 year in the early-TIPS and standard care group, respectively. No Significant heterogeneity was observed, when assessed mortality at 1 year (OR=0.64, 95%CI= 0.46-0.90, P=0.010; I^2^ = 38.4%, P=0.165) in **Figure 3**.

A subgroup analysis in **Table 2** by RCTs and non-RCTs demonstrated a significant difference in them (RCTs: OR=0.36, 95%CI=0.17-0.73, P=0.005, I^2^ = 0%, P=0.374; non-RCTs: OR=0.75, 95%CI=0.52-1.08, P=0.122; I^2^ = 27.2%, P=0.253). Furthermore, a significant reduction in 1-year mortality, according to different Child-Pugh classes, was also observed (Child-Pugh B: OR=0.34, 95%CI=0.25-0.58 P<0.001; I^2^ = 0%, P=0.470 and Child-Pugh C: OR=0.35, 95%CI=0.18-0.68, P=0.002; I^2^ = 10.1%, P=0.329) in **Figure 3**.

#### Long-Term Rebleeding (1 Year)

Five studies (Garcia-Pagan et al., 2010; Garcia-Pagan et al., 2013; Rudler et al., 2014; Lv et al., 2019a; Lv et al., 2019b) with an overall 34 (8.5%) and 442 (32.6%) recorded patients in early-TIPS and standard treatment, respectively. A significant decrease in rebleeding risk with moderate heterogeneity was observed in early-TIPS compared to standard care (OR=0.16, 95%CI= 0.07-0.36, P<0.001; I^2^ = 52.7%, P=0.076) in **Table 2**.

A subgroup analysis in **Table 2** demonstrated that there was no statistical significance in RCTs (OR=0.17, 95%CI=0.02-1.56, P=0.116, I^2^ = 73.3%, P=0.053). On the contrary, a significant decrease was observed in non-RCTs (OR=0.12, 95%CI=0.04-0.40, P=0.001; I^2^ = 57.1%, P=0.097).

#### Mortality Caused by Bleeding

Four studies (Garcia-Pagan et al., 2010; Rudler et al., 2014; Lv et al., 2019a; Lv et al., 2019b) reported this outcome with 1 (0.3%) and 95 (7.2%) patients who died in early-TIPS and standard therapy, respectively. A significant improvement was observed in patients treated with early-TIPS compared to standard care for preventing bleeding-related mortality during a 1 year follow up (OR=0.07, 95%CI=0.02-0.29, P<0.001; I^2^ = 0%, P=0.989) in **Table 2**.

A subgroup analysis in **Table 2** demonstrated that early-TIPS showed a prior protective effect with no heterogeneity in non-RCTs (OR=0.07, 95%CI=0.02-0.33, P=0.001; I^2^ = 0%, P=0.940). However, no difference was observed in RCTs (OR=0.07, 95%CI=0.004-1.40, P=0.083; I^2^=NA, P=NA).

#### Mortality Caused by Liver Failure

Five studies (Garcia-Pagan et al., 2010; Garcia-Pagan et al., 2013; Lv et al., 2019a; Lv et al., 2019b) reported this outcome with 33 (8.3%) and 82 (6.0%) patients in early-TIPS and standard therapy, respectively. No statistically significant difference was observed in early-TIPS compared to standard therapy (OR=1.33, 95%CI=0.84-2.12, P=0.222; I^2^ = 1%, P=0.400) in **Table 2**.

A subgroup analysis in **Table 2** demonstrated no difference in RCTs (OR=0.71, 95%CI=0.21-2.38, P=0.580; I^2^ = 7.2%, P=0.229) and non-RCTs (OR=1.50, 95%CI=0.91-2.46, P=0.111; I^2^ = 13.6%, P=0.314).

#### Liver Transplantation

Five studies (Garcia-Pagan et al., 2010; Garcia-Pagan et al., 2013; Rudler et al., 2014; Lv et al., 2019a; Lv et al., 2019b) reported this outcome with 36 (9.0%) and 88 (6.5%) patients in early-TIPS and standard therapy, respectively. There was no statistic difference in the two groups (OR=1.36, 95%CI=0.83-2.15, P=0.183; I^2^ = 0%, P=0.891) in **Table 2**.

A subgroup analysis in **Table 2** demonstrated that no significant difference was observed in RCTs (OR=1.66, 95%CI=0.39-6.94, P=0.491; I^2^ = 0%, P=0.668) and non-RCTs (OR=1.33, 95%CI=0.82-2.16, P=0.247; I^2^ = 0%, P=0.652).

#### New or Worsening Ascites

**Figure 4:**
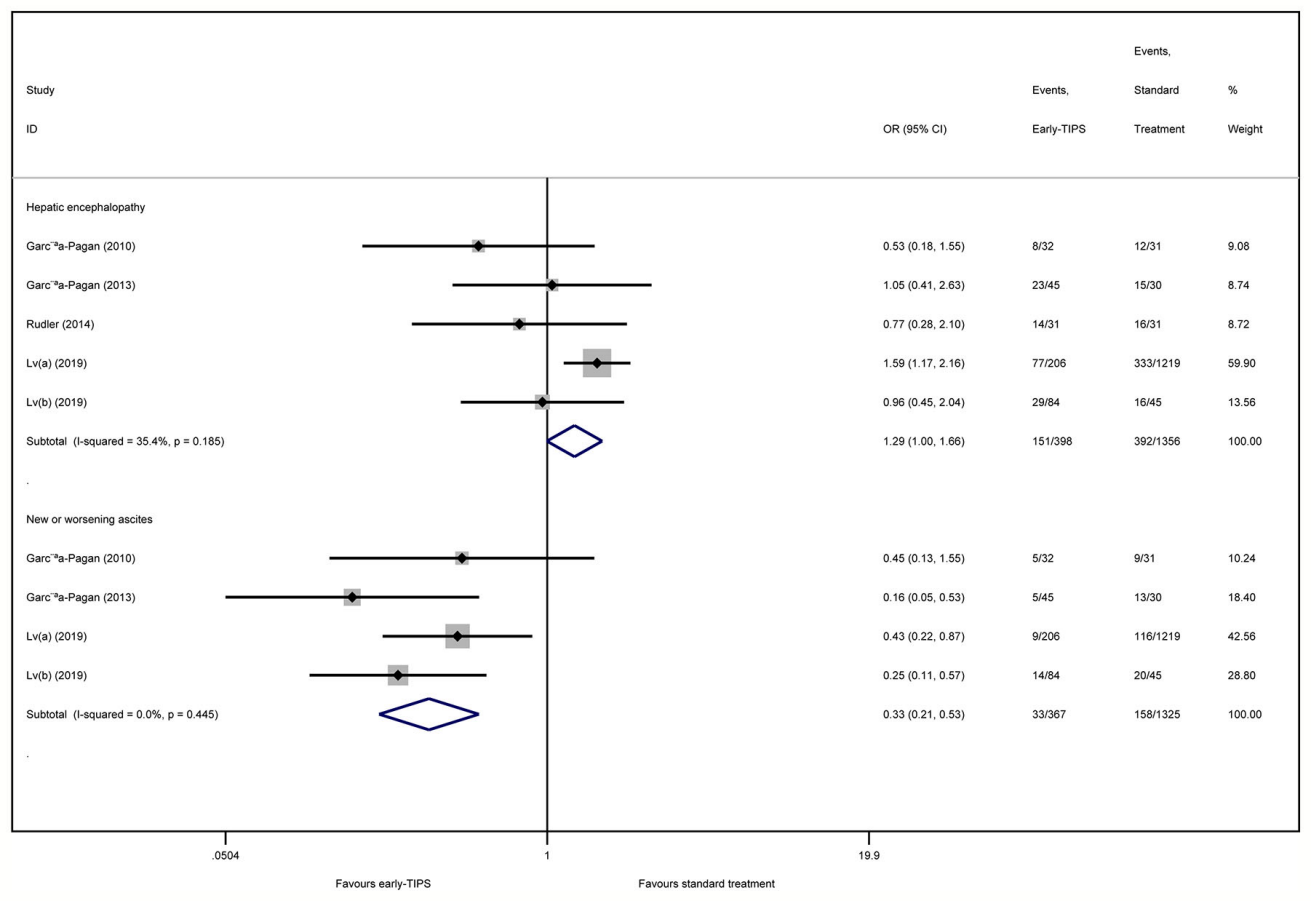
Forest plot demonstrating risk of hepatic encephalopathy and new or worsening ascites.

Four studies (Garcia-Pagan et al., 2010; Garcia-Pagan et al., 2013; Lv et al., 2019a; Lv et al., 2019b) reported this outcome, in which an overall 33 (9.0%) and 158 (11.9%) recoded patients suffered new or worsening ascites in early-TIPS and standard therapy, respectively. A significant lower risk of new or worsening ascites events was obtained in early-TIPS (OR=0.30, 95%CI= 0.21-0.53, P<0.001; I^2^ = 0%, P=0.445) in **Figure 4**.

A subgroup analysis in **Table 2** demonstrated a significant difference in RCTs (OR=0.30, 95%CI=0.15-0.60, P=0.001; I^2^ = 0%, P=0.431) and non-RCTs (OR=0.30, 95%CI=0.12-0.76, P=0.001; I^2^ = 49.6%, P=0.159).

### Sensitivity Analysis

No differences from the previous results were observed in the sensitivity analysis of excluding Child-Pugh A for three primary outcomes (treatment failure: OR=0.13, 95%CI=0.04-0.47, P=0.002; I^2^ = 0%, P=0.808, 6-weeks mortality: OR=0.07, 95%CI=0.02-0.33, P=0.001; I^2^ = 0%, P=0.925, and 6-weeks rebleeding: OR=0.2, 95%CI=0.10-0.40, P=0.007; I^2^ = 12.0%, P=0.321). In addition, the sensitivity analysis showed that early-TIPS would not increase the risk of hepatic encephalopathy (OR=1.80, 95%CI=0.66-4.92, P=0.253; I^2^ = 0%, P=0.900).

### Publication Bias

The Egger's test was carried out to detect publication bias in our outcomes, and demonstrated no publication bias in most of our outcomes, except in hepatic encephalopathy (P=0.003).

## Discussion

Despite the fact that almost 20% of patients suffered refractory bleeding or rebleeding and required a rescue TIPS procedure, standard therapy (pharmacotherapy *plus* endoscopic therapy) has been widely performed for decades, profiting from the obvious effect on hemostasis and especially immediate performance during endoscopic examination, while the variceal was actively bleeding. Based on current guidelines (Garcia-Tsao et al., 2017), studies exploring the role of early-TIPS in management of acute variceal bleeding and refining the target population that will benefit from this treatment, is indispensable in further research. We carried out this systematic review and meta-analysis, evaluating the effect on early-TIPS procedure in advanced cirrhosis patients with AVB compared to current standard therapy.

In this study, significant decreases in 6 weeks mortality and treatment failure (considered as the main outcomes by Baveno VI consensus (de Franchis, 2015)) were identified. These results were consistent with the AASLD guideline (Garcia-Tsao et al., 2017), in which considering early-TIPS could improve survival at 6 weeks and avoid treatment failure for AVB patients. Early-TIPS also showed a preventative effect on short-term rebleeding. The main determinant of improvement in survival (Rigau et al., 1989) and rebleeding risk is probably based on a higher rate of bleeding control which benefits form reduction in portal hypertension. Monescillo et al. found that more active bleeding at urgent endoscopy and a greater rate of treatment failure were always associated with higher portal hypertension, therefore, patients with a high risk of rebleeding and treatment failure could preferably avoid early rebleeding and treatment failure based on reduction in HVPG (Bosch and Garcia-Pagan, 2003) and have a higher rate of successful treatment (Chau et al., 1998; Azoulay et al., 2001) in the TIPS procedure. In the subgroup analysis, RCTs were associated with significantly higher improvement in 6-weeks survival and treatment failure in early-TIPS, which provided convincing evidence to support this outcome more effectively. Among these outcomes, a sensitivity analysis did not show a difference compared to previous results, which supported the reliability of this study more effectively.

As the most concerning complications, hepatic encephalopathy was not increased in the early-TIPS group, compared to standard therapy, which is consistent with previous a meta-analysis (Deltenre et al., 2015) and the AASLD guideline (Garcia-Tsao et al., 2017) that considers no difference between the two interventions. Nevertheless, early-TIPS improved the risk of encephalopathy in non-RCTs. From five included studies, only one study by Lv et al. (2019b) showed an increased effect on encephalopathy, while the others showed no difference between the two interventions. Additionally, the highest OR occurred in Lv et al., 2019a, in which patients with Child-Pugh A were enrolled, which might be a predictive factor for overt hepatic encephalopathy (Hassoun et al., 2001). Therefore, the performance of early-TIPS remains controversial. The other possible predictors, such as a higher patient age (Stefankova et al., 2007), being female (Hassoun et al., 2001), a high Child-Pugh score (ter Borg et al., 2004), indication for TIPS, hepatic arterial blood flow changes after TIPS, or type of stent, have been discussed in other studies (Pomier-Layrargues et al., 2001; Angeloni et al., 2004; Amarapurkar et al., 2006). In addition, we also performed a sensitivity analysis without including Child-Pugh A patients, which demonstrated that early-TIPS would not increase the risk of hepatic encephalopathy. Child-Pugh A patients were normally in a compensated stage, which means that the liver function was acceptable for their body. The Child-Pugh A patients were barely suffering hepatic encephalopathy before the placement of early-TIPS, however, the placement of early-TIPS directed blood ammonia which could have moved through the liver without metabolizing, therefore we preferred to object to performing TIPS in patients with a Child-Pugh A classification. The potential publication bias was detected in this outcome, which might suggest the presence of language bias, or a lack of publication of small trials with opposing results. In addition, both RCTs and non-RCTs in the subgroup analysis showed no difference between the interventions, however, considering the pooled negative effect of including all studies, we assumed that this uncertainty might due to the scarcity of studies, therefore, newer and richer knowledge is required to prevent this considerable complication in further research. Another main complication which affected long-term survival was ascites (Garcia-Tsao et al., 2017), which showed a significantly lower risk in early-TIPS in our study, and which profited from the reduction in HVPG.

Long-term effects on early-TIPS demonstrated that 1-year survival in our study was certainly improved compared to standard therapy. Among these five included studies, RCTs (Garcia-Pagan et al., 2010; Lv et al., 2019a) identified a significant improvement in 1-year survival in patients who received early-TIPS, which came down to the same conclusion in a previous meta-analysis (Deltenre et al., 2015). In addition, we assessed the relative risk of bleeding-related death, which demonstrated a significant reduction in bleeding-related death in the early-TIPS group, and also supports the previously mentioned conclusion that TIPS played a key role in decreasing HVPG (Bosch and Garcia-Pagan, 2003). Therefore, this protective effect on improving long-term survival might be due to the reduction of bleeding-related death compared to standard therapy. The early-TIPS was also associated with lower 1-year rebleeding risk, which is consistent with previous studies (Deltenre et al., 2015). Nevertheless, RCTs showed no difference between the two interventions, which might suggest that the lack of studies affect the accuracy of the results. This issue could be solved in future by increased studies on this subject.

No difference between the two interventions was observed in patients who successfully underwent liver transplantation and might account for the fact that early-TIPS would not increase the risk of death due to liver failure. However, more patients in early-TIPS underwent liver transplantation. Deltenre et al. (2015) considered that this outcome might be due to the reduction in treatment failure and rebleeding, which allows some patients with severe cirrhosis, who should not have met the criteria for transplantation, to use standard therapy and who could be enrolled in a transplantation list. In consideration of the improvement in treatment failure and short-term survival, without increasing liver failure death in early-TIPS patients, we assumed that the performance of early-TIPS could be an alternative treatment choice, to rescue more AVB patients by undergoing further liver transplantation. However, without addressing the global shortage of organ donations, this advantage is bound to be limited.

The most essential purpose for studies on treatment of AVB are to refine the target population that might benefit from early-TIPS (Garcia-Tsao et al., 2017). In other studies, patients with Child-Pugh B and C were equally associated with higher survival in the early-TIPS group, which was consistent with the previous study (Deltenre et al., 2015) and AASLD guideline (Garcia-Tsao et al., 2017). Although previous research considered that the reason there was no difference in terms of mortality in Child-Pugh B and Child-Pugh C patients, might be due a limited sample size, however, the result remained unchanged in this study, which suggests that patients with decompensated cirrhosis and AVB could consider receiving early-TIPS either in Child-Pugh B or Child-Pugh C, which is strongly supported in the current recommendation of the European Association for the Study of the Liver (EASL) clinical practice guidelines (EASL, 2018) of 2018. Nevertheless, in an observational study (Hernandez-Gea et al., 2019), patients with Child-Pugh B with AVB demonstrated a low mortality, and early-TIPS did not improve the survival compared to standard care (survival at 6 weeks: 94% vs. 90%; survival at 1 year: 77% vs. 75%). However, the rescue-TIPS was performed in their standard group, which might potentially improve the final outcomes of survival. Therefore, there is still no clear evidence that use of early-TIPS for AVB patients with Child-Pugh B is a predicting factor for bad prognosis. At present, early-TIPS is only recommended in high risk patients (Child-Pugh C and Child-Pugh B with acute variceal bleeding). Nevertheless, Lv et al. (2019b) considered that high risk patients only made up a small percentage of AVB, therefore, patients with Child-Pugh A and Child-Pugh B without AVB, were investigated in their study and it was found that in Child-Pugh A patients, the mortality in the standard treatment group was already low and no difference was observed compared to early-TIPS; in Child-Pugh B patients, improvement in survival was only observed in patients with AVB, and there was no benefit in survival in Child-Pugh B patients without AVB. As mentioned previously, the patients with Child-Pugh A might suffer a higher risk of encephalopathy compared to standard therapy. In conclusion, whether non-high-risk patients could benefit from early-TIPS requires further investigative research.

The advantages of our study are as follows: compared to the previous studies, we reported serval new endpoints and drew some new conclusion with several updated studies: (1) mortality and rebleeding rate at 6-weeks, which was considered the primary endpoint for studies for treatment of AVB, showed a significant reduction in early-TIPS; (2) early-TIPS does not increase risk of liver transplantation, which might provide evidence that AVB patients, who may die using standard therapy, could survive and eventually undergo liver transplantation; (3) early-TIPS showed a lower risk of bleeding-related death and new or worsening ascites, which could testify to the effect of reducing HVPG; (4) early-TIPS does not increase the risk of hepatic encephalopathy. We also assessed the long-term survival and subgroup analysis by Child-Pugh C and Child-Pugh B classification, which offered better evidence for clinicians to select patients who are appropriate for early-TIPS. Finally, a subgroup analysis with RCTs was performed to provide better evidence for uncertainties (long-term survival, hepatic encephalopathy and target population) on the latest clinical guidelines.

### Limitation

Among the five included studies, four were published by two authors (Lv and Garcia-Pagan), which indicated that a large population came from only these two studies which might create a potential bias. Additionally, the scarcity of included studies and the deficiency in valuable outcomes should be considered as a limitation in this study, which might affect the accuracy of the results.

## Conclusions

This meta-analysis revealed that in patients with AVB in cirrhosis, early covered-TIPS could rescue more patients, demonstrating a significant improvement in treatment failure, both in short- and long-term mortality, rebleeding risk, and new or worsening ascites compared to current standard therapy. The early-covered-TIPS could be recommended in the management of high-risk AVB patients with cirrhosis, except for patients with Child-Pugh A. In consideration of the scarcity of current evidence, further researches exploring solutions to prevent hepatic encephalopathy and refining selection criteria for early-covered-TIPS are required.

## Author Contributions

The study was designed by CZ and G-QZ. SL, JL, QW, H-XZ and L-LL contributed data to the paper. Statistical analysis and interpretation of data were performed by Y-MN and SL. The response of review comments and language adjustment were performed by G-QZ and A-LZ. All authors were involved in drafting and revision of the manuscript for important intellectual content and approved the final version to be published.

## Funding

This study was supported by the Science and technology projects of Songjiang District, Shanghai (No. 19sjkjgg142). The funders had no roles in study design, data collection and analysis, decision to publish, or preparation of the manuscript.

## Conflict of Interest

The authors declare that the research was conducted in the absence of any commercial or financial relationships that could be construed as a potential conflict of interest.
