# Supplementary material for: Early-TIPS Versus Current Standard Therapy for Acute Variceal Bleeding in Cirrhosis Patients: A Systemic Review With Meta-analysis
Source: Front Pharmacol. 2020 May 20;11:603. doi: 10.3389/fphar.2020.00603 (PMC7282546; doi:10.3389/fphar.2020.00603)
Supplement: Supplementary file 1 [file DataSheet_1.pdf]

Supplementary Material

Supplemental Table 1 Reason for exclusion of literature

Study	Year	Title	Reasons
Trebicka J	2018	Does Transjugular Intrahepatic Portosystemic Shunt Stent Differentially Improve Survival in a Subset of Cirrhotic Patients	The study design wasn't meet the inclusion criteria.
Bucsics	2017	Re-bleeding rates and survival after early transjugular intrahepatic portosystemic shunt (TIPS) in clinical practice	The study design wasn't meet the inclusion criteria.
Njei	2017	Early TIPS in U.S. Patients Hospitalized with Acute Esophageal Variceal Bleeding	The study design wasn't meet the inclusion criteria.
Bureau	2016	Transjugular Intrahepatic Portosystemic Shunts With Covered Stents Increase Transplant-free Survival of Patients With Cirrhosis and Recurrent Ascites	The study design wasn't meet the inclusion criteria.
Luo	2015	advanced cirrhosis combined with Portal Vein Thrombosis: A Randomized Trial of TIPS versus Endoscopic Band Ligation Plus Propranolol for the Prevention of Recurrent Esophageal Variceal Bleeding1	The study design wasn't meet the inclusion criteria.
Perarnau	2014	Covered vs. uncovered stents for transjugular intrahepatic portosystemic shunt: A randomized controlled trial	The study design wasn't meet the inclusion criteria.
Orloff	2014	Fifty-three Years' Experience With Randomized Clinical Trials of Emergency Portacaval Shunt for Bleeding Esophageal Varices in Cirrhosis 1958-2011	The study design wasn't meet the inclusion criteria.
Deipolyi	2014	Reduction in Portal Venous Pressure by Transjugular Intrahepatic Portosystemic Shunt for Treatment of Hemorrhagic Stomal Varices	The population wasn't eligible.
Siramopliwat	2014	Transjugular intrahepatic portosystemic shunts and portal hypertension-related complications.	The study design wasn't meet the inclusion criteria.
Gonzalez	2011	Adding banding ligation is effective as rescue therapy to prevention variceal rebleeding in haemodynamic non-responders to pharmacological therapy.	The study design wasn't meet the inclusion criteria.
Afdhal	2010	Early TIPS to Improve Survival in Acute Variceal Bleeding	The study design wasn't meet the inclusion criteria.
Bosch	2009	Pharmacological Versus Endoscopic Therapy in the Prevention of Variceal Hemorrhage: And the Winner Is	The study design wasn't meet the inclusion criteria.

Biecker	2007	Prognostic role of the initial portal pressure gradient reduction after TIPS in patients with cirrhosis	The study design wasn't meet the inclusion criteria.
Franchis	2006	Endoscopy Critics vs. Endoscopy Enthusiasts for Primary Prophylaxis of Variceal Bleeding	The study design wasn't meet the inclusion criteria.
Monescillo	2004	Influence of Portal Hypertension and Its Early Decompression by TIPS Placement on the Outcome of Variceal Bleeding	The study design wasn't meet the inclusion criteria.
Ogusu	2002	Endoscopic injection sclerotherapy for esophageal varices in cirrhotic patients without hepatocellular carcinoma: a comparison of longterm survival between prophylactic therapy and emergency therapy	The study design wasn't meet the inclusion criteria.
Gulberg	2002	Transjugular Intrahepatic Portosystemic Shunting is not Superior to Endoscopic Variceal Band Ligation for Prevention of Variceal Rebleeding in Cirrhotic Patients: a Randomized, Controlled Trial	The study design wasn't meet the inclusion criteria.
Gøtzsche PC	2000	Somatostatin analogues for acute bleeding esophageal varices	The study design wasn't meet the inclusion criteria.
Villarreal	1998	Transjugular Intrahepatic Portosystemic Shunt Versus Endoscopic Sclerotherapy for the Prevention of Variceal Rebleeding After Recent Variceal Hemorrhage	The study design wasn't meet the inclusion criteria.
Daniel Alvarez	1997	Daily Variation in Portal Blood Flow and the Effect of Propranolol Administration in a Randomized Study of Patients With Cirrhosis	The population wasn't eligible.
Mario,A	1997	Effects of Isosorbide-5-mononitrate Compared With Propranolol on First Bleeding and Long-term Survival in Cirrhosis	The study design wasn't meet the inclusion criteria.
Sauer	1997	Transjugular Intrahepatic Portosystemic Stent Shunt Versus Sclerotherapy Plus Propranolol for Variceal Rebleeding	The study design wasn't meet the inclusion criteria.
Jalan	1997	A Randomized Trial Comparing Transjugular Intrahepatic Portosystemic Stent-Shunt With Variceal Band Ligation in the Prevention of Rebleeding From Esophageal Varices	The study design wasn't meet the inclusion criteria.
Cabrera J	1996	Endoscopic versus TIPS Therapy for the Prevention of variceal Hemorrhage: Is TIPS tops	The study design wasn't meet the inclusion criteria.
Faust, F	1996	Double-Blind Randomized Controlled Trial Comparing Terlipressin and Somatostatin for Acute Variceal Hemorrhage	The study design wasn't meet the inclusion criteria.
Cabrera J	1996	Transjugular Intrahepatic Portosystemic Shunt Versus Sclerotherapy in the Elective Treatment of Variceal Hemorrhage	The study design wasn't meet the inclusion criteria.
Ink, O	1994	Has Propranolol Rendered Sclerotherapy Obsolete for Poor Risk Alcoholic Cirrhotic Patients?	The study design wasn't meet the inclusion criteria.

Gin-Ho Lo	1994	Endoscopic injection sclerotherapy vs. endoscopic variceal ligation in arresting acute variceal bleeding for patients with advanced hepatocellular carcinoma	The population wasn't eligible.
Pramod, G	1994	Role of Omeprazole in Prevention and Treatment of Postendoscopic Variceal Sclerotherapy Esophageal Complications	The study design wasn't meet the inclusion criteria.
Gin-Ho Lo	1993	Does propranolol maintain post-sclerotherapy variceal obliteration? A prospective randomized study	The population wasn't eligible.
D.Bbargava	1992	Prospective Randomized Comparison of Sodium Tetradecyl Sulfate and Polidoeanol as Variceal Sclerosing Agents	The study design wasn't meet the inclusion criteria.
Shinn-Jang Hwang	1992	A randomized controlled trial comparing octreotide and vasopressin in the control of acute esophageal variceal bleeding	The study design wasn't meet the inclusion criteria.
Acob, K	1991	Hemodynamic Events in a Prospective Randomized Trial of Propranolol Versus Placebo in the Prevention of a First Variceal Hemorrhage	The study design wasn't meet the inclusion criteria.
Y Chawla	1990	Studies in Sclerotherapy: Comparison of Sodium Tetradecyl Sulphate(STS) with Absolute Alcohol(AA) as Sclerosants in the Treatment of Esophageal Varices	The study design wasn't meet the inclusion criteria.
S.Kitano	1989	Human thrombin plus 5 per cent ethanolamine oleate injected to scleroseoesophageal varices: a prospective randomized trial	The study design wasn't meet the inclusion criteria.
O'connor	1989	Comparison of Three Nonsurgical Treatments for Bleeding Esophageal Varices	The study design wasn't meet the inclusion criteria.
S.Kitano	1988	Trial of sclerosing agents in patients with esophageal varices	The study design wasn't meet the inclusion criteria.
Harold, C	1969	Prophylactic Portacaval Anastomosis in Cirrhotic Patients with Esophageal Varices and Ascites	The study design wasn't meet the inclusion criteria.

Supplemental Table 2 Quality assessment of included non-RCT studies, using Newcastle-Ottawa Scale

			Selection				Comparability		Outcomes			
**Author**	**Year**	**Study design**	**Representativeness of the exposed cohort**	**Selection of the non exposed cohort**	**Ascertainment of exposure**	**Demonstration that outcome of interest was not present at start of study**	**Comparability of cohorts on the basis of the design or analysis**		**Assessment of outcome**	**Was follow-up long enough for outcomes to occur**	**Adequacy of follow up of cohorts**	**Total**
Garcia-Pagan	2013	Cohort study	1	1	1	0	1	1	1	1	1	**8**
Rudler	2014	Cohort study	1	1	1	0	1	1	1	1	1	**8**
Lv	2018	Cohort study	1	1	1	0	1	1	1	1	1	**8**

Supplemental Table 3 Quality assessment of risk of bias in included RCT, using the Cochrane Collaboration tool

Study	Random sequence generation (Selection bias)	Allocation concealment (Selection bias)	Blinding of participants and personnel (Performance bias)	Blinding of outcome assessment (Detection bias)	Incomplete outcome data (Attrition bias)	Selective reporting (Reporting bias)	Other bias
García-Pagan, 2010	Low	Low	Low	Low	Low	Low	Unclear
Lv, 2019	Low	Low	Low	Low	Low	Low	Unclear
